# Treatment via oral midodrine in a patient with prolonged hypotension following carotid artery stenting: a case report

**DOI:** 10.1186/s13256-022-03270-5

**Published:** 2022-02-09

**Authors:** Yaser Jenab, Kaveh Hosseini, Seyed-Mohammad Abtahi-Eivary

**Affiliations:** 1 Fellowship of Interventional Cardiology, Tehran Heart Center, Tehran University of Medical Sciences, Tehran, Islamic Republic of Iran; 2 Tehran Heart Center, Tehran University of Medical Sciences, Tehran, Islamic Republic of Iran; 3 Tehran University of Medical Sciences, Tehran, Islamic Republic of Iran

**Keywords:** Carotid artery stenosis, Carotid artery stenting, Hypotension, Midodrine, Case report

## Abstract

**Background:**

Hypotension and bradycardia are common hemodynamic complications following carotid artery stenting in patients with carotid artery stenosis. Intravenous fluid resuscitation and inotropes such as dopamine are conventional treatments for post-carotid artery stenting hypotension. However, in case of resistant hypotension, there is no clear treatment method. In this report, while intravenous fluid and inotropes did not resolve the patient’s hypotension, oral midodrine treated post-carotid artery stenting hypotension.

**Case presentation:**

In this report, we present an 82-year-old Caucasian man complaining of a single episode of unilateral visual loss. The patient had left internal carotid artery stenosis and underwent carotid artery stenting. After the procedure, he developed prolonged post-carotid artery stenting hypotension, which was resistant to intravenous fluids and inotropes but immediately showed a promising response to oral midodrine.

**Conclusion:**

Oral midodrine can be considered in treatment of post-carotid artery stenting hemodynamic complications.

## Background

Carotid artery stenosis is an atherosclerotic condition that is more prevalent in people older than 80 years old (7.5% of men, 5% of women) [1]⁠ and is responsible for about 10–15% of cerebral ischemic strokes [2]⁠⁠. Carotid endarterectomy (CEA) and carotid artery stenting (CAS) are two major treatments for carotid stenosis, reducing the risk of stroke in these patients [3]⁠. CEA is usually the treatment of choice but in certain conditions such as previous neck surgery, unfavorable carotid anatomy, history of neck radiation, and high-risk patients for surgery, CAS is preferred [4]⁠.

Hypotension, bradycardia, and asystole are hemodynamic complications of CAS that occur mostly during and up to 12 hours after the procedure. Studies show that 5–47% of patients undergoing CAS may develop hemodynamic complications [5–9]. Asymptomatic stenosis, stenosis proximity to bifurcation, calcification at the bifurcation, eccentric stenosis, and dilation percentage may predict the risk of hemodynamic complications of CAS [10, 11]. Post-CAS hypotension correlates with higher risk of stroke/transient ischemic attack (TIA), myocardial infarction (MI), prolonged hospital stay (> 1 day), and in-hospital mortality [12]⁠.

In case of CAS-related hypotension, intravenous fluid resuscitation and vasopressors (for example, dopamine, phenylephrine, and norepinephrine) are usual treatments, but some studies have shown the effectiveness of oral midodrine, an alpha-adrenergic agonist, in treating these hemodynamic complications [13, 14]⁠.

In this article, we present a 82-year-old man with symptomatic carotid stenosis and prolonged CAS-related hypotension who was treated with oral midodrine.

## Case presentation

The patient was a 82-year-old Caucasian man with a past medical history of dyslipidemia and smoking, who complained of acute unilateral visual loss (Amaurosis Fugax) for 20 minutes, 10 days ago. On admission, his vital signs were stable: blood pressure 110/80 mmHg and pulse rate of 60 beats per minute (bpm).

**Fig. 1:**
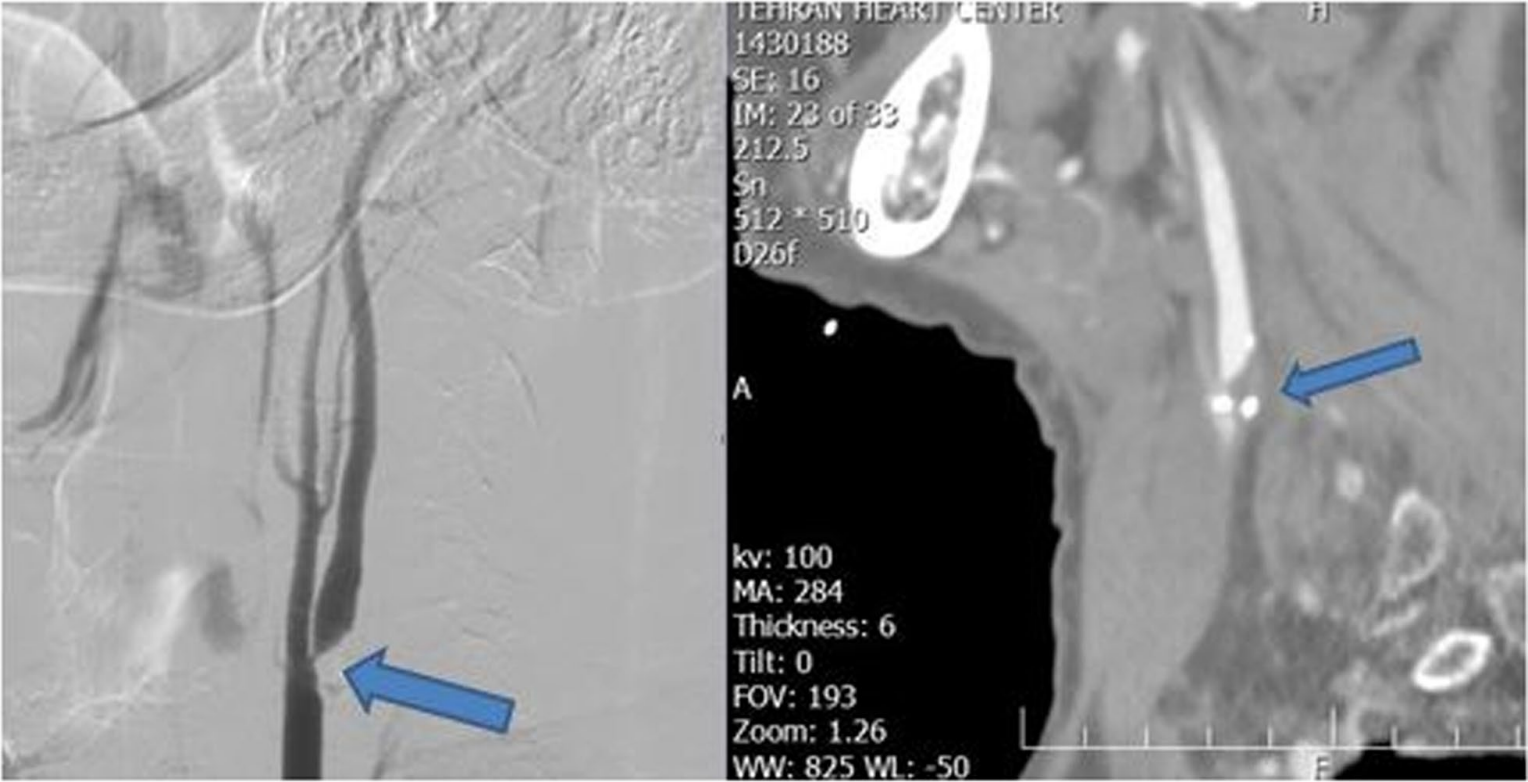
Before stenting: Left image, severe stenosis in origin of left internal carotid artery. Right image, CT angiography and calcification of LICA

**Fig. 2:**
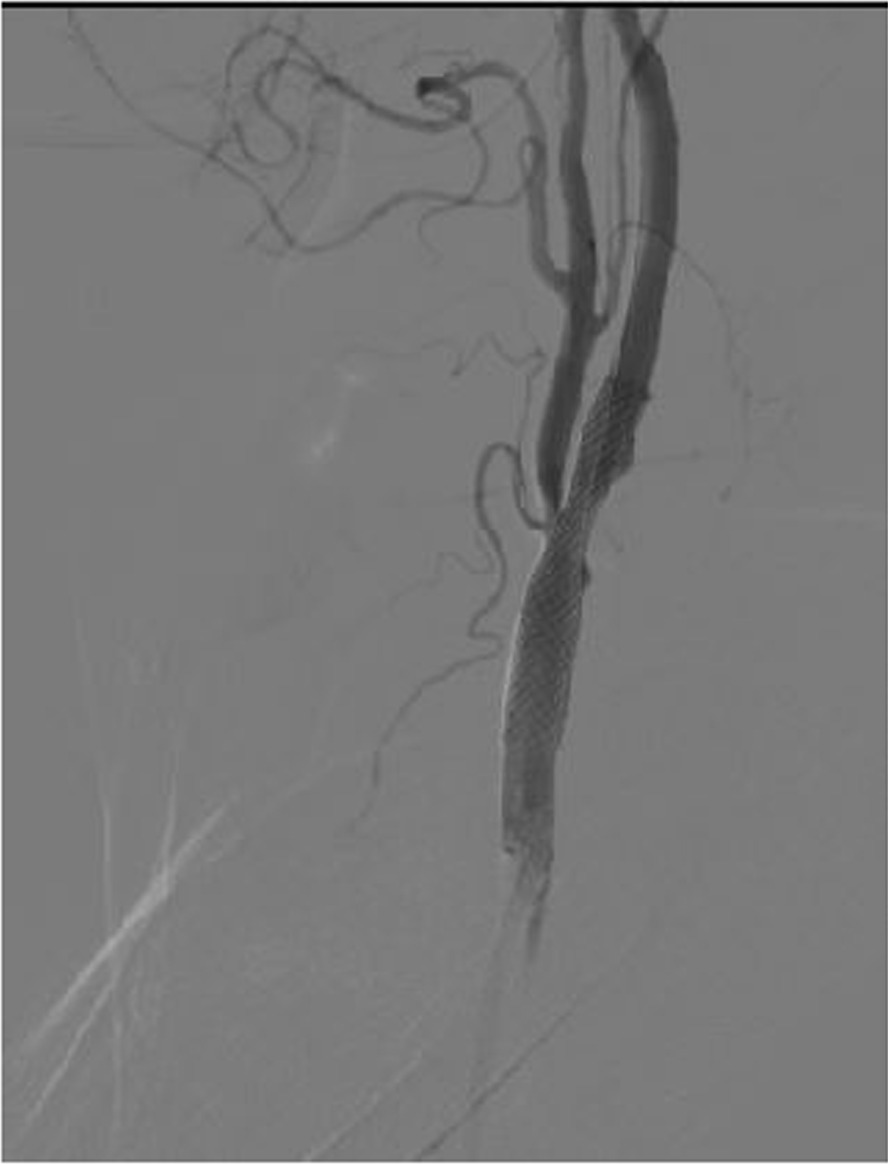
After stenting: Successful percutaneous transluminal angioplasty of LICA

On transcranial Doppler sonography (TCD), severe stenosis of left internal carotid artery (LICA) was noted. Computed tomography (CT) angiography confirmed severe stenosis of LICA with some calcifications (Fig. 1). As the patient refused CEA, he was planned for carotid artery stenting. Conventional angiography with digital subtraction angiography (DSA) showed severe stenosis, of LICA origin, Fig. 1. The day after admission, carotid artery stent implantation (Wall stent 7–30) with the FilterWire EZ Embolic Protection System and postdilation with Viatrac 5–20 at 8 atm was done (Fig. 2). During stent postdilatation, the patient developed bradycardia and his blood pressure decreased to 80/50 mmHg. He was given a 0.6 mg dose of atropine intravenously and intravenous fluids, with subsequent correction of the bradycardia and hypotension. The patient was transferred to the ward uneventfully. However, the patient had recurrent hypotension afterwards, which was unresponsive to fluid boluses. Serum therapy with normal saline was not effective and dopamine was initiated because the patient complained of dizziness. However, 3 days after the procedure, the patient was still hypotensive and symptomatic. Midodrine 15 mg every 6 hours was added to his prescription. After a few hours, his blood pressure increased to 111/78 mmHg and dopamine was discontinued. The next day, midodrine was discontinued and the patient was discharged without hypotension or any other symptoms.

## Discussion and conclusion

Carotid artery interventions such as carotid artery stenting (CAS) and carotid endarterectomy (CEA) sometimes cause hypotension and bradycardia. The hemodynamic depression caused by CAS is a result of baroreceptor activity of the carotid body. The carotid body is a small organ near the carotid bifurcation that consists of baroreceptors and chemoreceptors that controls blood pressure, heart rate, and oxygen saturation. Normally, hypertension stretches the carotid body, resulting in carotid baroreceptor stimulation and, therefore, inhibition of sympathetic and stimulation of parasympathetic outflow of CNS into the heart and peripheral vessels. This is called the arterial baroreflex, which decreases heart rate and blood pressure [15]⁠.

Some studies have investigated the risk factors of developing hemodynamic depression after CAS. These include proximity of stenosis to carotid bifurcation, presence of calcified plaque, length of stenosis, asymptomatic stenosis, balloon-to-artery ratio, concurrent contralateral stenosis, eccentric (versus concentric) stenosis, atherosclerotic (versus restenotic) lesion, female gender, positive stress test, age > 70, history of MI or angina, and an urgent (versus elective) procedure [6, 8, 10–12]⁠⁠. These hemodynamic complications of CAS are associated with a higher risk of postoperative stroke/TIA, MI, chronic heart failure (CHF), dysrhythmia, prolonged length of stay, and in-hospital mortality [6, 12]⁠. Furthermore, the CAS-related hypotension might be persistent, possibly due to the continued stretching of the carotid sinus by the self-expanding stent [14]⁠.

Usual treatments of CAS-related hemodynamic depression includes intravenous fluid resuscitation and intravenous inotropes such as dopamine and norepinephrine. Usually, these treatments are effective, but in case of unfavorable response, other treatments such as oral midodrine have been suggested [14]⁠.

In the present case, we showed that oral midodrine is an effective treatment for prolonged and resistant post-CAS hypotension. Midodrine is an alpha-1 adrenergic agonist, which stimulates vasoconstriction and increases blood pressure. Midodrine is used mostly to treat orthostatic hypotension [16, 17]⁠⁠. Winters *et al*. reported a case of CAS-related hemodynamic depression in 2014, which was resistant to fluid resuscitation and metaraminol infusion, and lasted more than 1 week. In this case, treatment with oral midodrine was effective [18]⁠. Sharma *et al*. reported four cases of post-CAS hypotension treated with oral midodrine. The author concluded that oral midodrine was well tolerated and as effective as an intravenous dopamine infusion [14]⁠.

Finally, although the evidence on the use of oral midodrine in post-CAS hypotension is limited, it seems that oral midodrine is effective and should be considered as a treatment alternative in these patients.

## Data Availability

Not applicable.

## Abbreviations

CAS: Carotid artery stenting
CEA: Carotid endarterectomy
TCD: Transcranial Doppler sonography
LICA: Left internal carotid artery
